# Effects of Vitamin D on Blood Pressure, Arterial Stiffness, and Cardiac Function in Older People After 1 Year: BEST‐D (Biochemical Efficacy and Safety Trial of Vitamin D)

**DOI:** 10.1161/JAHA.117.005707

**Published:** 2017-10-24

**Authors:** Joseph Tomson, Harold Hin, Jonathan Emberson, Rijo Kurien, Michael Lay, Jolyon Cox, Michael Hill, Linda Arnold, Paul Leeson, Jane Armitage, Robert Clarke

**Affiliations:** ^1^ Clinical Trial Service Unit and Epidemiological Studies Unit Nuffield Department of Population Health University of Oxford United Kingdom; ^2^ MRC Population Health Research Unit Nuffield Department of Population Health University of Oxford United Kingdom; ^3^ Hightown Surgery Banbury Oxfordshire United Kingdom; ^4^ Oxford Cardiovascular Clinical Research Facility Radcliffe Department of Medicine John Radcliffe Hospital Oxford United Kingdom

**Keywords:** blood pressure, cardiac dysfunction, vitamin D, Cardiovascular Disease, Epidemiology, Aging

## Abstract

**Background:**

The relevance of vitamin D for prevention of cardiovascular disease is uncertain. The BEST‐D (Biochemical Efficacy and Safety Trial of vitamin D) trial previously reported effects of vitamin D on plasma markers of vitamin D status, and the present report describes the effects on blood pressure, heart rate, arterial stiffness, and cardiac function.

**Methods and Results:**

This was a randomized, double‐blind, placebo‐controlled trial of 305 older people living in United Kingdom, who were allocated vitamin D 4000 IU (100 μg), vitamin D 2000 IU (50 μg), or placebo daily. Primary outcomes were plasma concentrations of 25‐hydroxy‐vitamin D and secondary outcomes were blood pressure, heart rate, and arterial stiffness in all participants at 6 and 12 months, plasma N‐terminal prohormone of brain natriuretic peptide levels in all participants at 12 months, and echocardiographic measures of cardiac function in a randomly selected subset (n=177) at 12 months. Mean (SE) plasma 25‐hydroxy‐vitamin D concentrations were 50 (SE 2) nmol/L at baseline and increased to 137 (2.4), 102 (2.4), and 53 (2.4) nmol/L after 12 months in those allocated 4000 IU/d, 2000 IU/d of vitamin D, or placebo, respectively. Allocation to vitamin D had no significant effect on mean levels of blood pressure, heart rate, or arterial stiffness at either 6 or 12 months, nor on any echocardiographic measures of cardiac function, or plasma N‐terminal prohormone of brain natriuretic peptide concentration at 12 months.

**Conclusions:**

The absence of any significant effect of vitamin D on blood pressure, arterial stiffness, or cardiac function suggests that any beneficial effects of vitamin D on cardiovascular disease are unlikely to be mediated through these mechanisms.

**Clinical Trial Registration:**

URL: https://www.clinicaltrialsregister.eu/ctr-search/search. Unique identifier: EudraCT number: 2011–005763‐24a

**Clinical Perspective**

**What Is New?**

- This randomized trial of 305 community‐dwelling older people demonstrated that supplementation with high‐dose vitamin D compared with placebo for 12 months had no significant effect on blood pressure, arterial stiffness, echocardiographic measures of cardiac function, or on biochemical markers of cardiac function.

**What Are the Clinical Implications?**

- Pending the results of ongoing large trials of vitamin D for the prevention of fracture and other disease outcomes, the absence of any significant effects of vitamin D on blood pressure, arterial stiffness, or cardiac function observed in the present study provides no support for use of vitamin D supplements for the prevention of cardiovascular disease.

## Introduction

Prospective studies have reported inverse associations of plasma concentrations of 25‐hydroxy‐vitamin D (25[OH]D) with the risk of all‐cause and cardiovascular disease (CVD) mortality with an approximately linear association on a logarithmic scale up to ≈90 nmol/L,^1^, ^2^, ^3^, ^4^ but substantial uncertainty persists about the causal relevance of low vitamin D concentrations for CVD.^5^, ^6^ It is possible that the inverse associations of plasma 25(OH)D concentrations with CVD may be caused by confounding by other aspects of lifestyle, and any such effects cannot be entirely excluded in observational studies. Randomized trials are needed to establish causality, but older trials were neither designed to specifically evaluate CVD outcomes, nor used sufficient doses of vitamin D to achieve plasma 25(OH)D concentrations associated with the lowest risks of CVD demonstrated in these observational studies.^6^

The mechanisms underlying the associations of vitamin D with CVD are not fully understood,^7^ but previous studies have reported inverse associations of plasma 25(OH)D concentrations with hypertension^8^ and arterial stiffness.^9^ Mendelian randomization studies have reported lower levels of systolic blood pressure among those with genetically lower plasma 25(OH)D concentrations, but the effect sizes of genetically determined differences were very small.^10^ Randomized trials of vitamin D and meta‐analyses of trials have shown no significant effect on blood pressure^11^ or arterial stiffness.^12^, ^13^ It is unclear whether the null results reflect small sample sizes, insufficient dosing of vitamin D, or the relatively short duration of treatment in such trials. Furthermore, low plasma 25(OH)D concentrations have also been associated with heart failure, and may be predictive of poorer outcomes in patients with heart failure,^14^, ^15^, ^16^ but whether supplementation with vitamin D improves cardiac function remains unclear.

The BEST‐D (Biochemical Efficacy and Safety Trial of vitamin D) trial evaluated the safety and efficacy of daily supplementation with high‐dose vitamin D (4000 IU or 2000 IU daily) or placebo for 1 year among older people living in Oxfordshire, United Kingdom, as a pilot study for a large trial of vitamin D for prevention of CVD, fracture, and cancer.^17^, ^18^ The results of the primary outcomes of the BEST‐D trial of the effects of supplementation with vitamin D versus placebo on plasma concentrations of 25(OH)D and other plasma markers of vitamin D status have been previously published.^18^ The aim of the present report was to describe the effects of supplementation with vitamin D versus placebo on prespecified secondary outcomes of blood pressure, heart rate, and arterial stiffness after 6 and 12 months of treatment; on plasma concentrations of N‐terminal prohormone of brain natriuretic peptide (NT‐proBNP) at 12 months; and on echocardiographic measures recorded at 12 months in a randomly selected subset of 177 participants.

## Methods

### Study Design and Recruitment

**Figure 1:**
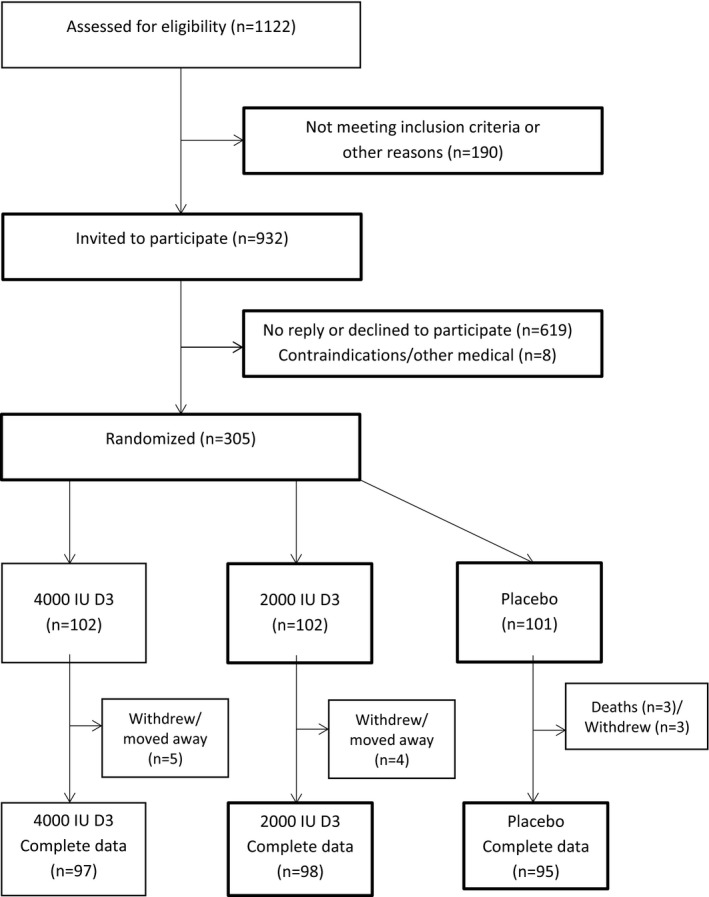
CONSORT flow diagram for BEST‐D trial. BEST‐D indicates Biochemical Efficacy and Safety Trial of vitamin D; CONSORT, Consolidated Standards of Reporting Trials.

The study design and the results of the effects of supplementation with vitamin D on plasma concentrations of 25(OH)D and related biochemical markers and other measures in the BEST‐D trial have been previously reported.^17^, ^18^ Briefly, participants aged 65 years or older living in the community were recruited by an invitation letter from a single general practice in Banbury, Oxfordshire, United Kingdom, and had to be ambulatory, living in the community, and not taking more than 400 IU (10 μg) vitamin D daily (Figure 1). Trained research nurses visited the study participants in their homes at baseline, 6, and 12 months. Information was recorded directly into bespoke electronic case report forms and included medical history, presence of vascular risk factors, and medication use. Blood samples were collected and physical measurements recorded as previously described.^17^, ^18^ Study visits were scheduled at the same time of the day to minimize any effects of diurnal variation and participants were advised to avoid alcohol, tea, and coffee before each visit. At the 6‐ and 12‐month visits, compliance with study medications, serious adverse events, and nonserious adverse events leading to discontinuation of study treatment were recorded. During the baseline visit, telephone randomization allocated participants to study treatment (vitamin D 4000 IU or 2000 IU or placebo daily) in a double‐blind manner. The minimization algorithm included age, body mass index, smoking history, ethnicity, and history of fracture as variables. Vitamin D3 and the matching placebo soft‐gel capsules were provided by Tischcon Corporation (Westbury, NY, USA) and packaged in child‐proof containers. All trial investigators, trial staff, and participants were blinded to treatment allocation until the data analysis plan was finalized and the database locked for analysis after trial completion. All participants provided written informed consent to a protocol that was approved by the Multicentre Research Ethics Service Committee (Oxford B), United Kingdom, and complied with the Declaration of Helsinki.

### Blood Sample Collection and Biochemical Measurements

Blood samples were collected at each visit into vacutainers containing either lithium heparin or EDTA, processed within 4 hours, and stored at −80°C for further analyses. Plasma concentrations of 25(OH)D were measured using an Access 2 immunoassay analyzer (Beckman Coulter Ltd, High Wycombe, United Kingdom). Plasma concentrations of NT‐proBNP were measured using an immunoassay with electro‐chemiluminescent labels (human NT‐proBNP standard kit, Meso Scale Diagnostics, Rockville, MD, USA) and reading plates on a Sector Imager 6000. Quality control material was run in duplicate with a between‐run precision of 18% for NT‐proBNP at 180.5 pg/mL.

### Blood Pressure and Arterial Stiffness

Blood pressure and arterial stiffness were measured at each visit after 10 minutes of rest in the seated position. First, a finger probe (Pulsetrace PCA 2) was placed on the right forefinger to record the digital volume pulse trace using photoplethysmography over 30 to 60 s.^19^ This was followed by blood pressure and brachial artery arterial stiffness measurements made over 2 minutes using a TensioClinicTM^®^ Arteriograph.^20^ The systolic and diastolic blood pressure, heart rate, aortic pulse wave velocity, and augmentation index were recorded. The Arteriograph recorded the pressure waveforms at the brachial artery using an oscillometric method after occluding blood flow locally by raising the brachial cuff pressure to 35 mm Hg above the systolic pressure. The upper edge of the cuff detected the pressure changes over 8 s and calculated the pulse wave velocity (pulse wave velocity=D/[RT/2], where the jugulo‐symphyseal distance [D] was the approximate length of the descending aorta, divided by the time interval [RT] between the peaks of the forward and reflected waves). Augmentation index reflected the augmented pressure in the central aorta caused by the reflected wave form, expressed as a percentage of central pulse pressure (Augmentation index=100 [P2–P1]/PP), where P1 was the amplitude of the forward wave, P2 was the amplitude of the reflected wave, and PP was the pulse pressure. The stiffness index and reflective index were additional measures of peripheral arterial function derived from the Pulsetrace device. The stiffness index was calculated as participant's height/PPT, where PPT was the time between the peaks of the forward and reflected waves measured using the Pulsetrace device, and the reflective index was calculated as the height of the forward wave divided by the height of the reflected wave.

### Echocardiographic Measures

From among those randomized and willing to have an echocardiogram, 177 people were randomly selected for echocardiography (ensuring participants were also proportionally selected by month of recruitment). Echocardiographic measurements were recorded at a single center by a cardiologist or trained echocardiography sonographer within 2 weeks of the scheduled final visit. Examinations were conducted using a Philips CX50 ultrasound system. After removing clothing above the waist, participants were asked to lie in the left lateral position with a 30° to 45° elevation of the headrest. Standard image acquisition parasternal (long and short axes) and apical (2‐ and 4‐chamber) views were obtained. For analyses, 3 consecutive cardiac cycle loops were recorded at end‐expiration. Ejection fraction (measured by Biplane Simpson's or M‐mode), fractional shortening, (M‐mode), and chamber dimensions (2‐dimensional imaging) were measured. Images were processed using TomTec Imaging Systems software for the outcome measures of left ventricular (LV) systolic function (ejection fraction, fractional shortening, and global longitudinal strain) and diastolic function (E/E^I^, LV internal dimension, LV end‐diastolic volume, and left atrial [LA] dimension and LA volume). All dimensions and volumes were corrected for body surface area recorded at randomization. Global longitudinal strain estimates were adjusted for heart rate and systolic blood pressure recorded at the time of the examination.

### Statistical Analyses

The prespecified co‐primary outcomes were mean plasma concentration of 25(OH)D at 12 months and the percentage of participants with 12‐month plasma 25(OH)D concentration >90 nmol/L.^17^ Prespecified secondary outcomes included 6‐ and 12‐month measures of blood pressure, heart rate, and arterial stiffness, and 12‐month concentration of NT‐proBNP. Values for NT‐proBNP were log‐transformed for all analyses. Participants randomly selected for echocardiography at 12 months had 3 measures of systolic function (ejection fraction, fractional shortening, and global longitudinal strain) and 5 measures of diastolic function (E/E^I^, LV internal dimension, LV end‐diastolic volume, LA dimension, and LA volume) recorded. It was prespecified that secondary outcomes would be compared between those allocated vitamin D 4000 IU/d and those allocated placebo, between those allocated 2000 IU/d and those allocated placebo, between the 2 active vitamin D doses, and between those allocated either dose of vitamin D versus placebo.^17^ The primary method of analysis was an analysis of covariance adjusted for the participant's baseline value (which provides a superior test to a comparison of mean follow‐up levels or a comparison of mean changes from baseline).^21^ Since echocardiographic measures were only recorded at the final visit, comparisons between allocated treatment groups were conducted using analysis of variance with missing data imputed using multiple imputation within allocated groups using Rubin's rules.^22^ Analyses were conducted according to the intention‐to‐treat principle and all *P* values were 2‐sided and considered statistically significant at the 5% level (but allowance for multiple testing was made in the interpretation of results). Analyses were conducted independently of the sources of support using SAS version 9.3 and R version 2.11.1.

## Results

### Baseline Characteristics

From September 24, 2012, 305 participants were randomly allocated over a 6‐month period to take either 4000 IU D (n=102) or 2000 IU D (n=102), or placebo (n=101) daily for 12 months (Figure 1). Baseline characteristics by allocated treatment are shown in Table 1. Arteriograph measures were missing for 16% of participants at baseline and 6 months, and 14% of participants at 12 months. Pulse trace measures were missing for 13% of participants at baseline, 16% at 6 months, and 22% at 12 months. Systolic blood pressure at randomization was strongly correlated with aortic augmentation index (*r*=0.41), but more weakly correlated with pulse wave velocity (*r*=0.24), stiffness index (*r*=0.19), and reflection index (*r*=0.17) (Table 2). There was no correlation of either the stiffness or the reflection index measured by the PulseTrace with the pulse wave velocity measured by the Arteriograph (*r*=0.01 and −0.08, respectively). None of these baseline vascular measures correlated even moderately with plasma concentrations of 25(OH)D.

**Table 1:** Participant Characteristics by Allocated Treatment

	4000 IU/d (n=102)	2000 IU/d (n=102)	Placebo (n=101)
Age, y	71 (6)	72 (6)	72 (6)
Male	51%	50%	51%
Current smoker	7%	7%	7%
Prior disease			
Heart disease^a^	20%	11%	11%
Stroke/TIA	5%	8%	6%
Hypertension	39%	43%	35%
Diabetes mellitus	9%	9%	9%
Medication			
Antihypertensives	49%	51%	46%
Statin	31%	28%	23%
Antithrombotic	20%	23%	18%
Vitamin D (≤400 IU/d)	12%	10%	13%
Calcium	4%	1%	4%
Physical measurements			
Height, cm	168 (10)	168 (10)	167 (10)
Weight, kg	77 (17)	78 (15)	79 (15)
Body mass index, kg/m^2^	27 (5)	27 (4)	28 (5)
Body surface area, m^2^	1.88 (0.25)	1.90 (0.23)	1.91 (0.22)
Arteriograph measurements			
Systolic blood pressure, mm Hg	132.7 (21.1)	131.8 (17.1)	129.5 (18.8)
Diastolic blood pressure, mm Hg	78.0 (11.3)	76.6 (10.3)	76.6 (12.1)
Heart rate, beats/min	66.2 (10.5)	66.1 (12.1)	64.9 (8.8)
Pulse wave velocity, m/s	10.0 (1.9)	9.6 (1.6)	9.7 (1.8)
Aortic augmentation index, %	37.7 (16.0)	36.8 (14.4)	36.0 (15.5)
Pulse trace measurements			
Stiffness index, m/s	9.2 (2.3)	9.1 (2.4)	9.5 (2.8)
Reflection index, m/s	64.3 (14.0)	63.1 (15.4)	66.9 (12.5)

Mean (SD) or % shown. TIA indicates transient ischemic attack.

a Defined as heart attack, angina, or heart failure.

**Table 2:** Correlations Between Particular Baseline Measurements

	SBP	DBP	HR	PWV	AAI	SI	RI	25(OH)D
Systolic blood pressure	1.00	0.76	−0.01	0.24	0.41	0.19	0.17	−0.03
Diastolic blood pressure	···	1.00	0.00	0.21	0.30	0.30	0.27	0.00
Heart rate	···	···	1.00	0.13	−0.46	0.08	−0.32	0.01
Pulse wave velocity	···	···	···	1.00	0.30	0.01	−0.08	−0.07
Aortic augmentation index	···	···	···	···	1.00	−0.13	0.08	0.00
Stiffness index	···	···	···	···	···	1.00	0.67	0.01
Reflection index	···	···	···	···	···	···	1.00	0.03
25(OH)D	···	···	···	···	···	···	···	1.00

AAI indicates aortic augmentation index; DBP, diastolic blood pressure; HR, heart rate; PWV, pulse wave velocity; RI, reflection index; SBP, systolic blood pressure; SI, stiffness index.

The self‐correlation of values recorded within individuals at 0 and 12 months in the placebo‐allocated group was 0.74 for systolic blood pressure, 0.69 for diastolic blood pressure, 0.68 for heart rate, 0.72 for pulse wave velocity, and 0.65 for augmentation index, but only 0.50 for stiffness index and 0.45 for reflection index. By contrast, the self‐correlation for plasma 25(OH)D concentrations over 12 months in the placebo group was 0.90.

### Effects on Plasma 25(OH)D Concentrations

**Figure 2:**
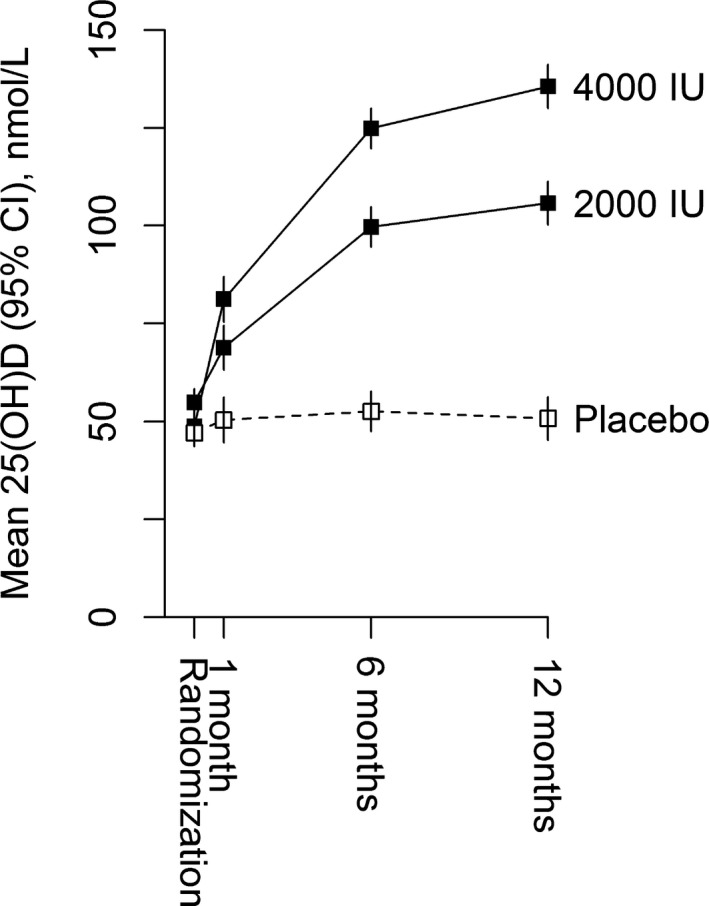
Effect of allocation to vitamin D vs placebo on mean plasma 25(OH)D concentrations. Values shown are unadjusted means (95% confidence intervals).

Compliance across the 3 treatment arms was comparable. Among those allocated to 4000 IU, 2000 IU, or placebo, 93%, 93%, and 87%, respectively, reported taking their capsules on all or most days at 6 months, while 90%, 92%, and 85% reported doing so at the 12‐month visit. Mean (SE) plasma 25(OH)D concentrations were 50 (SE 2) nmol/L at baseline and increased to 137 (2.4), 102 (2.4), and 53 (2.4) nmol/L after 12 months of treatment in those allocated 4000 IU/d, 2000 IU/d of vitamin D, or placebo, respectively (Figure 2).

### Effects on Blood Pressure and Arterial Stiffness

Table 3 shows mean (SE) levels of blood pressure and arterial stiffness at 6 and 12 months adjusted for baseline levels. When compared with placebo, neither dose of vitamin D (or both doses combined: data not shown) had any significant effect on systolic or diastolic blood pressure, heart rate, aortic augmentation index, stiffness index, or reflection index at either time point. Allocation to vitamin D 4000 IU/d was associated with marginally higher pulse wave velocity at 12 months compared with placebo (10.1 versus 9.6 m/s, *P*=0.009 before accounting for multiple testing).

**Table 3:** Effect of Allocation to Vitamin D on Blood Pressure and Arterial Stiffness After 6 and 12 Months of Treatment

	4000 IU/d (n=102)	2000 IU/d (n=102)	Placebo (n=101)	*P* Value^a^	*P* Value^b^	*P* Value^c^
Systolic blood pressure, mm Hg						
6 mo	129.7 (1.31)	129.9 (1.36)	127.8 (1.44)	0.34	0.29	0.90
12 mo	132.5 (1.43)	131.8 (1.51)	131.8 (1.51)	0.71	0.98	0.73
Diastolic blood pressure, mm Hg						
6 mo	75.9 (0.91)	76.5 (0.95)	75.5 (1.02)	0.81	0.49	0.63
12 mo	77.2 (0.89)	77.0 (0.94)	76.6 (0.96)	0.65	0.73	0.92
Heart rate, beats/min						
6 mo	64.9 (0.81)	65.3 (0.85)	66.7 (0.92)	0.14	0.27	0.71
12 mo	66.0 (0.84)	66.4 (0.87)	67.0 (0.87)	0.40	0.64	0.72
Pulse wave velocity, m/s						
6 mo	9.8 (0.13)	9.9 (0.13)	9.9 (0.13)	0.46	0.89	0.55
12 mo	10.1 (0.13)	9.9 (0.14)	9.6 (0.14)	0.009	0.20	0.19
Aortic augmentation index, %						
6 mo	36.7 (1.34)	37.5 (1.38)	35.0 (1.33)	0.36	0.19	0.68
12 mo	37.5 (1.28)	36.8 (1.36)	37.1 (1.38)	0.83	0.86	0.69
Pulse trace stiffness index, m/s						
6 mo	9.6 (0.25)	9.6 (0.24)	9.5 (0.24)	0.70	0.85	0.83
12 mo	9.4 (0.28)	9.4 (0.27)	9.5 (0.36)	0.94	0.98	0.96
Pulse trace reflection index, m/s						
6 mo	64.6 (1.40)	65.4 (1.31)	65.2 (1.34)	0.75	0.89	0.66
12 mo	66.3 (2.17)	68.4 (2.53)	66.3 (2.32)	0.98	0.54	0.52

Values shown are mean (SE) adjusted for the baseline value. Missing data were imputed using multiple imputation.

a *P* value comparing 4000 IU daily vs placebo.

b *P* value comparing 2000 IU daily vs placebo.

c *P* value comparing 4000 vs 2000 IU daily.

### Effects on Echocardiographic Measures of Cardiac Function and on Brain Natriuretic Peptide

Of the 177 participants selected for echocardiographic measures, 44 (15% among those allocated vitamin D and 14% among those allocated placebo) did not attend the appointment for the following reasons: an inability to schedule a visit within 2 weeks of the final visit (n=39), they had died or moved away (n=3), or missed their appointment (n=2). Among those who did attend, levels of ejection fraction were strongly positively correlated with fractional shortening (*r*=0.72) and inversely correlated with LV internal dimension (*r*=−0.32) and LA volume (*r*=−0.28) (Table 4). None of the echocardiographic measures were strongly correlated with plasma concentration of 25(OH)D or NT‐proBNP (Table 4).

**Table 4:** Correlations Between 12‐Month Measurements of Echocardiographic Parameters, 25(OH)D Concentration, and Ln NT‐proBNP Concentration

	EF	FS	GLS	E/E′	LVID	LVEDV	LAD	LAV	25(OH)D	Ln NT‐proBNP
Ejection fraction	1.00	0.72	−0.18	0.07	−0.32	−0.29	−0.16	−0.28	0.04	0.02
Fractional shortening	···	1.00	−0.17	0.11	−0.29	−0.31	−0.10	−0.25	0.00	0.07
Global longitudinal strain	···	···	1.00	−0.03	0.10	0.20	0.19	0.11	−0.08	−0.04
E/E^I^	···	···	···	1.00	−0.14	−0.15	−0.10	−0.04	−0.07	0.13
LV internal dimension	···	···	···	···	1.00	0.76	0.32	0.48	0.00	0.04
LV end‐diastolic volume	···	···	···	···	···	1.00	0.46	0.49	−0.02	−0.02
LA dimension	···	···	···	···	···	···	1.00	0.50	−0.16	0.14
LA volume	···	···	···	···	···	···	···	1.00	−0.20	0.12
25(OH)D	···	···	···	···	···	···	···	···	1.00	0.04
Ln NT‐proBNP	···	···	···	···	···	···	···	···	···	1.00

E/E^I^ indicates ratio of early peak inflow velocity to early diastolic peak lateral mitral annular velocity; EF, ejection fraction; FS, fractional shortening; GLS, global longitudinal strain; LAD, left atrial dimension; LAV, left atrial volume; LVEDV, left ventricular end‐diastolic volume; LVID, left ventricular internal dimension; NT‐proBNP, N‐terminal prohormone of brain natriuretic peptide.

Allocation to either dose of vitamin D compared with placebo had no significant effect on any measure of either systolic or diastolic function at 12 months (Table 5). Likewise, allocation to either dose of vitamin D had no significant effect on plasma concentration of Ln NT‐ProBNP (6.1 [0.1] versus 6.2 [0.1] Ln pg/mL; *P*=0.11: Table 5). Even in post‐hoc analyses among the subgroup with an ejection fraction of <50%, there was no suggestion of any beneficial effect of vitamin D supplementation (data not shown).

**Table 5:** Effect of Allocation to Vitamin D3 (4000 IU or 2000 IU Daily) Versus Placebo on Echocardiographic Measures of Systolic and Diastolic Cardiac Function After 12 Months of Treatment and on Plasma Concentrations of NT‐proBNP

	Either Dose of Vitamin D	Placebo	*P* Value
Systolic function			
Ejection fraction, %	60.5 (1.1)	60.8 (1.7)	0.89
Fractional shortening, %	30.8 (1.1)	30.8 (1.5)	0.98
Global longitudinal strain, % a	−19.9 (0.8)	−19.5 (1.17)	0.73
Diastolic function			
E/E^I^	7.2 (0.3)	7.8 (0.4)	0.23
LV internal dimension, cm^b^	4.8 (0.1)	4.7 (0.1)	0.65
LV end‐diastolic volume, mL/m²^b^	105.3 (3.4)	106.2 (4.7)	0.88
LA dimension, cm^b^	3.8 (0.1)	3.9 (0.1)	0.28
LA volume, mL/m^2^ b	44.2 (2.0)	41.7 (2.9)	0.47
Ln NT‐proBNP, ln pg/mL	6.1 (0.1)	6.2 (0.1)	0.11

Values shown are mean (SE). Missing data were imputed using multiple imputation. Analyses of echocardiography (including missing data imputation) were limited to the subset of 177 participants (117 on either dose of vitamin D and 60 on placebo) who were randomly selected to have an echocardiogram at 12 mo and did not have artificial valves (2 patients) or severe aortic stenosis (1 patient) at the time of examination. E/E^I^ indicates ratio of early peak inflow velocity to early diastolic peak lateral mitral annular velocity; LV, left ventricular; NT‐proBNP, N‐terminal prohormone of brain natriuretic peptide; SBP, systolic blood pressure.

a Adjusted for heart rate and SBP at the time of examination.

b Adjusted for body surface area at randomization.

### Sensitivity Analyses

For all outcomes, results of all treatment comparisons were not materially altered when restricted to participants with no missing data (ie, complete case analyses: data not shown).

## Discussion

Despite allocation to vitamin D achieving and maintaining plasma 25(OH)D concentrations more than 2‐fold greater than those allocated to placebo for 1 year, supplementation with high‐dose vitamin D did not have any significant effect on systolic or diastolic blood pressure or on measures of central or peripheral arterial stiffness. Similarly, vitamin D had no effect on any echocardiographic measures of systolic or diastolic cardiac function or on biochemical markers of cardiac function.

While these results for the effects of vitamin D on blood pressure and arterial stiffness are consistent with the evidence from previous meta‐analysis of randomized trials evaluating effects of vitamin D on blood pressure^23^ and arterial stiffness,^13^ the present trial assessed the effects of both higher doses of vitamin D and longer duration of treatment than most of the previous trials addressing these questions. A meta‐analysis of previously published trials, including individual participant data from 27 other trials involving 3092 participants, demonstrated no effect of vitamin D supplementation on blood pressure (systolic blood pressure −0.5 mm Hg; 95% confidence interval, −1.3 to 0.4; *P*=0.27).^23^

Likewise, previous trials assessing the effects of supplementation with vitamin D on cardiac function have reported conflicting results. The PRIMO (Paricalcitol Capsule Benefits in Renal Failure ‐ Induced Cardiac Morbidity) trial^24^ (n=227) in patients with chronic kidney disease reported that calcitriol treatment for 48 weeks had no beneficial effects on cardiac function. The VINDICATE (Vitamin D Treating Patients with Chronic Heart Failure) trial^25^ (n=229), which involved patients with heart failure and a mean ejection fraction of 26%, reported that 4000 IU vitamin D daily for 12 months increased LV ejection fraction by 6% (95% confidence interval, 3.20–8.95, *P*<0.0001) and reduced LV end‐systolic or diastolic volume, although there was no impact on 6‐minute walking distance, which was the prespecified primary end point. However, the present trial with echocardiographic measures in 177 healthy older participants and mean ejection fraction of 60% demonstrated no beneficial effects of vitamin D on any echocardiographic measures of systolic or diastolic function or on global longitudinal strain, although for this population, the mean LV function was in the normal range.

While the limitations of the Arteriograph and Pulsetrace devices for assessing arterial stiffness are acknowledged (reflected by the lower self‐correlations compared with standard blood pressure measures),^26^ the absence of effects of high‐dose vitamin D on blood pressure and arterial stiffness observed in this report does not support supplementation with vitamin D for CVD prevention being mediated through any of these mechanisms. Nevertheless, this study cannot exclude benefits for CVD prevention beyond 1 year of treatment, or those mediated through other mechanisms. The absence of effects of vitamin D on cardiac function, including assessments of strain imaging, may also reflect the relatively healthy participant cohort and their stable cardiac function as the mean blood pressure and LV ejection fraction were within the expected normal range for this age group.

The mean plasma 25(OH)D concentrations in this population were also similar to those of healthy adults living in the United Kingdom.^2^ However, despite the lack of demonstrable effect in this study, the effects of vitamin D supplementation in those with lower mean plasma 25(OH)D concentrations or pre‐existing heart failure cannot be entirely excluded. Over 95% of the participants reported having European ancestry and, hence, the study was unable to assess any differential effects of vitamin D in other ethnic groups. Published reports of completed large trials assessing the effects of vitamin D on incident cardiovascular events are difficult to interpret given the relatively modest doses of vitamin D (400–800 IU daily) assessed.^27^, ^28^ However, ongoing large trials, involving over 56 000 participants, are currently assessing the effects of much higher doses of vitamin D on CVD and other disease outcomes.^29^, ^30^, ^31^ For example, these trials are testing vitamin D doses of 2000 IU daily (VITAL (Vitamin D and Omega‐3 Trial): n=25 897), 60 000 IU monthly (D‐Health (D‐Health Trial: a randomised trial of vitamin D for prevention of mortality an cancer): n=20 000) and 60 000 IU monthly (TIPS‐3 (International Polycap Study 3): n=5500). Recently, the ViDA (Vitamin D Assessment Study) trial also reported no beneficial effects of supplementation with vitamin D (administered as 100 000 IU monthly for 3.3 years) on CVD outcome in 5108 participants.^32^ Given the lack of any beneficial effects on CVD outcomes in several randomized‐controlled trials conducted to date, and additional uncertainty about the relevance of vitamin D for incidence of diabetes mellitus,^33^, ^34^ the results of ongoing and future trials of vitamin D supplementation are required to address the effects of vitamin D on incident CVD outcomes, in addition to effects on fracture and other disease outcomes, before advocating use of vitamin D supplements for prevention of CVD.^35^

## Sources of Funding

This work was supported by the British Heart Foundation, the BHF Centre of Research Excellence and Clinical Trial Service Unit, Nuffield Department of Population Health, University of Oxford, Oxford, United Kingdom.

## Disclosures

None.
